# Antimicrobial and Antibiofilm Potential of Acyclic Amines and Diamines against Multi-Drug Resistant *Staphylococcus aureus*

**DOI:** 10.3389/fmicb.2017.01767

**Published:** 2017-09-15

**Authors:** Gurmeet Kaur, P. Balamurugan, Sahana Vasudevan, Saikiran Jadav, S. A. Princy

**Affiliations:** Quorum Sensing Laboratory, Centre for Research in Infectious Diseases, School of Chemical and Biotechnology, SASTRA University Thanjavur, India

**Keywords:** biofilm, *Staphylococcus aureus*, multi-drug resistance, antibiofilm, diamines, antibacterial

## Abstract

Multi-drug resistant *Staphylococcus aureus* (MDRSA) remains a great challenge despite a decade of research on antimicrobial compounds against their infections. In the present study, various acyclic amines and diamines were chemically synthesized and tested for their antimicrobial as well as antibiofilm activity against MDRSA. Among all the synthesized compounds, an acyclic diamine, (2,2′-((butane-1,4-diylbis(azanediyl)bis(methylene))diphenol) designated as ADM 3, showed better antimicrobial activity (minimum inhibitory concentration at 50 μg/mL) and antibiofilm activity (MBIC_50_ at 5 μg/mL). In addition, ADM 3 was capable of reducing the virulence factors expression (anti-virulence). Confocal laser scanning microscope analysis of the *in vitro* tested urinary catheters showed biofilm reduction as well as bacterial killing by ADM 3. On the whole, our data suggest that acyclic diamines, especially ADM 3 can be a potent lead for the further studies in alternative therapeutic approaches.

## Introduction

*Staphylococcus aureus*, a Gram-positive, facultative anaerobic cocci bacterium, is one of the most notorious pathogen, causing infections in humans. Their abilities to evade the host immune defense mechanism and resistance to the first and second generation antibiotics has made the pathogen a subject of interest in the scientific community (Fedtke et al., 2004). *S. aureus* is an opportunistic pathogen related to the various types of infectious diseases such as wound infections, catheter-related bloodstream infections (CRBSI), musculoskeletal infections, toxic shock syndrome and, about 20% of population worldwide found to be the long-term carrier as a part of their normal flora (Kluytmans et al., 1997; Cole et al., 2001). Various factors associated with *S. aureus* such as virulence gene expressions, cell to cell signaling mechanism, inactivation of antibiotics, alteration in target sites, efflux pumps, and biofilm formation have led to the emergence of multi-drug resistant *S. aureus* (MDRSA) (Dinges et al., 2000; Becker et al., 2003; Zhu et al., 2008; Arya et al., 2011; Qayyum et al., 2016).

Planktonic microbes attach to a particular substratum and produce an anchoring polymer called as an extracellular polysaccharide (EPS) which leads to the formation of the multicellular microbial community known as biofilm (Flemming et al., 2007). The altered metabolic activity of the cells that are associated with biofilm formation have high rates of EPS production, activation of specific genes associated with biofilm formation and virulence, reduction in the growth rate than their planktonic counterparts (Flemming et al., 2007). Biofilm formation accounts for one of the major reasons for the emergence of multi-drug resistance (MDR) in various pathogenic microbes and is common in bacteria. In the biofilm mode of lifestyle, bacterial population acquires adaptation to tolerate and survive a wide range of diverse environmental stress such as scarce nutritional availability, antibiotics exposure, competition for survival in a multi-species environment (Parsek and Singh, 2003). The antibiotics tolerance ability of the biofilm cells complicates the treatment of various infections in humans, such as, cystic fibrosis, endocarditis, which includes biofilm formation on various biological implants such as, urinary catheters, heart catheters, various joint implants, and replacement of heart valves (Singh et al., 2000). Biofilms pose a threat to the human race because of their persistent nature and plays a major role in certain pathogenic infections (Singh et al., 2000; Ravichandiran et al., 2012; Arya and Princy, 2013; Otto, 2013). Several incidences have been reported with MDRSA strain infections such as CRBSI which is primarily due to either bacterial colonization on a device that may be intraluminal, i.e., formation of biofilm inside the lumen and causing persistent infection (Safdar and Maki, 2004). Similarly, musculoskeletal infections, wound infections, and nosocomial infections have also been reported by MDRSA which are very difficult to treat with the existing drugs (Bascones-Martinez et al., 2011; Abad et al., 2014; Vincze et al., 2014). Antibiofilm compounds are one such alternative option in the recent research focus. These small chemical ligands can independently inhibit bacterial biofilm or disrupt the biofilm matrix at the molecular level via, disturbing their metabolic pathways.

Medicinally important chemical compounds play a crucial role in treating various diseases which pose a threat to human survival at times. From our earlier reports, we have understood that acyclic amines could be promising leads against *S. aureus*, as one of our compounds (SarABI-12, 2-[(methylamino)methyl]phenol) was shown to have target specific interaction with the staphylococcal accessory regulator, SarA (Arya and Princy, 2013). In addition, our *in vitro* studies (Balamurugan et al., 2017) have confirmed the antibiofilm and anti-virulence properties against clinical *S. aureus* strains. The data also showed a significant reduction in the expression of SarA regulated virulence genes like *fnbA*, *hla*, and *hld*. It is also interesting to note that several researchers have reported the potential of cyclic amines and diamines to inhibit MDRSA, *Enterococcus* sp., *Clostridium difficile*, *Escherichia coli*, *Aspergillus oryzae*, *Aspergillus niger* (He et al., 2003; Abdel-Rahman et al., 2004; Hensler et al., 2006). The focus on the use of cyclic amines and diamines for antibacterial, antifungal and anti-proliferative activity has also been implicated (He et al., 2003; Juranić, 2014; Kazakova et al., 2014). Previous reports by Chtchigrovsky et al. (2013) shows the anti-proliferative activity of *trans-N*-heterocyclic carbene–amine–Pt(II) complexes. These complexes have the ability to bind to DNA, leading to intrastrand cross-links between two adjacent guanines and minor interstrand cross links, resulting in DNA damage and cell death (Chtchigrovsky et al., 2013). Similarly, Subík et al. (1977) report the antibacterial and antifungal activity of amine oxides due to their ability to disorganize the cellular membranes leading to cidal effects.

It is expected that similar to cyclic amines, acyclic amines can also have medicinally important biological potentials, supported by our previous works (Arya and Princy, 2013; Balamurugan et al., 2017). Hence, we have investigated the antibiofilm and antibacterial properties of acyclic amines which have not yet been explored against pathogenic bacteria.

## Materials and Methods

### Synthesis of Acyclic Amine and Diamine Compounds

**FIGURE 1:**
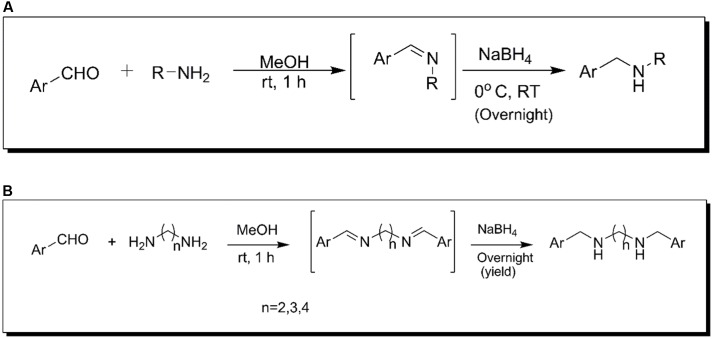
Schematic representation of the reaction scheme for synthesis of **(A)** acyclic amines **(B)** acyclic diamines.

The acyclic amines (AAM 1–5) and acyclic diamines (ADM 1–8) compounds were synthesized by a reductive amination process (Ramachandran et al., 2010). The synthesis process of acyclic amine and diamines is shown in the **Figures 1A,B**. For the synthesis of acyclic amines, a solution of the suitable aryl aldehyde (10 mmol) and the suitable acyclic amine (12 mmol) in methanol (5 mL) was stirred at room temperature for 1 h. The reactants were shown in Supplementary Table S1. The intermediate imines were reduced to the corresponding acyclic amines and diamines by the addition of sodium borohydride (15 mmol) at the ice-cold condition. After stirring, the reaction mixture was incubated at room temperature overnight and further diluted with water and extracted thrice with dichloromethane. The mixed organic phases were dried with anhydrous sodium sulfate, evaporated to dryness under reduced pressure to obtain the acyclic amines and diamines. The structures of the synthesized products were confirmed by ^1^H-NMR spectroscopy.

### Bacterial Strains and Growth Conditions

Clinical isolates of *S. aureus* were received from the rejected urinary catheters, JSS Medical College Mysore with prior approval from the Institutional Ethical Committee (IEC/JSSMC/PG/1050/2015-16). The strains were initially subjected to screening for MDR against the antibiotics: levofloxacin, ampicillin, ciprofloxacin, and methicillin. The *S. aureus* strain (MDRSA - QSL2040) that showed resistance to methicillin as well as other tested antibiotics (**Table 1**) was used as a test strain and the *S. aureus* (ATCC 25923) was used as reference strain (Kumar et al., 2013; Khatkar et al., 2014). These strains were cultured in the cation-adjusted Mueller–Hinton broth (CAMHB) for the minimal drug dose response against the growth and bactericidal actions. For the biofilm assays, the tryptic soy broth (TSB) supplemented with 3% NaCl and 0.5% glucose was used as the biofilm medium.

**Table 1:** Antibiotic susceptibility profiling of the two strains used in the study.

Antibiotics	Strains used	
	
	ATCC 25923	QSL2040
Levofloxacin	Sensitive	Resistant
Ampicillin	Sensitive	Resistant
Ciprofloxacin	Sensitive	Resistant
Methicillin	Sensitive	Resistant
Amoxicillin	Sensitive	Resistant
Penicillin	Sensitive	Resistant
Kanamycin	Sensitive	Resistant
Gentamicin	Sensitive	Resistant
Clindamycin	Sensitive	Intermediate
Vancomycin	Sensitive	Resistant
Oxacillin	Sensitive	Intermediate


*Sensitivity/resistance pattern was concluded as per the CLSI guidelines.*

### Evaluation of Acyclic Amine and Diamine Compounds for MIC, MBC, and MBIC

The minimum inhibitory concentration (MIC), the minimum bactericidal concentration (MBC), and the minimum biofilm inhibitory concentrations (MBIC) of the acyclic amines and diamines were determined according to the Clinical and Laboratory Standards Institute (CLSI) with slight modifications. The *S. aureus* clinical isolate (MDRSA - QSL2040) was grown overnight in TSB. The overnight culture was diluted 1:100 in physiological saline and the bacterial suspensions were adjusted to a final inoculum size of 1 × 10^6^ cells (Issam et al., 2015). The adjusted inoculum was added to the wells of a 96-well microtiter plate containing twofold serial dilutions of the compound (varying concentrations from 2 to 400 μg/mL as individual sets keeping different high concentrations) in CAMHB medium. The plates were incubated at 37°C for 24 h. The setup was carried out as independent experiments (in triplicates) to calculate the effective concentration.

The optical density (OD_600_) was read immediately after inoculation and again after 18 h of incubation at 37°C, in a microtitre plate reader (iMark, Bio-Rad, Japan). The lowest concentration that inhibited growth when compared to the untreated control culture was taken as the MIC. Similarly, MBC was determined by counting the number of colonies (CFU/mL) after 24 h of incubation at 37°C and was defined as the lowest concentration at which the viable cells were reduced to a level by ≥90% in comparison to the untreated control cultures. All independent assays were carried out in triplicates.

For biofilm assay, diluted overnight culture (1:100) was inoculated in TSB and allowed to grow till it reached exponential growth phase (5 h). The culture was tested for 0.5 McFarland unit and followed by inoculation along with varying concentrations of the compounds (2–400 μg/mL) in 96-well microtitre plates. Plates were incubated for 24 h at 37°C without shaking. After incubation, the wells were washed twice with 200 μL of phosphate-buffered saline (PBS) gently to remove the non-adherent cells. Adherent cells in the biofilm were fixed by adding 200 μL of 100% methanol prior to staining with 200 μL of 0.2% (w/v) crystal violet (CV) for 20 min. The excess stain was washed twice with PBS and the plates were air dried. The bound CV in the air dried plates were eluted with 200 μL of 33% acetic acid. The biofilm was quantitatively determined by measuring the absorbance at OD_595 nm_ in a microtitre plate reader (iMark, Bio-Rad, Japan). The concentration at which the formation of biofilm is inhibited ≥50% when compared to the untreated control culture is defined as MBIC_50_ and ≥90% is MBIC_90_. All the assays were carried out in triplicates.

### Anti-virulence Assays

Among the synthesized compounds, ADM 3 was selected as it showed higher antibiofilm activity at minimal concentration. The compound, ADM 3 was tested against the clinical isolate of *S. aureus* with two sub-MBIC_50_ concentrations (i.e., ¼× MBIC_50_, ½× MBIC_50_) and at a higher MBIC_50_ concentration (2× MBIC_50_). *S. aureus* reference strain, untreated with ADM 3 was kept as control. After 24 h, the cultures were centrifuged at 6000 rpm for 10 min at 4°C and filter sterilized in a 0.22 μm filter paper to collect the cell free supernatant. The collected supernatant was stored at 4°C and further used to quantify hemolysin and protease (Pietrow et al., 2013) among the various secreted exotoxins by *S. aureus*.

### Hemolytic Assay

Hemolysin in the culture supernatant was quantified according to the procedure described earlier with slight modifications (Cheung and Otto, 2012). Briefly, 10 mL of sheep blood was centrifuged at 2400 rpm for 5 min and the pellet obtained was washed twice with 10 mL of PBS. Ten microliters of this erythrocyte suspension was incubated with the cell free supernatant for 1 h at 37°C. Finally, the incubated sample was centrifuged at 2400 rpm for 5 min. Erythrocyte suspension treated with 1% Triton X-100 was used as a positive control. The optical density of the supernatant was read at 540 nm. Water along with the erythrocyte suspension was considered as blank. The percentage (%) hemolysis was calculated using the following formula:

%Hemolysis=absorbance(sample)−absorbance(blank)/absorbance(positive⁢control)

### Proteolysis Assay

Quantitative estimation of the protease was carried out with azocasein assay. A total of 200 μL of the cell free supernatant was incubated with 800 μL of azocasein for 30 min at 37°C. To this, 1200 μL of 1% trichloroacetic acid was added to arrest the enzymatic reaction. The contents were incubated on ice for 30 min and centrifuged at 12,000 rpm for 5 min. To 1600 μL of the supernatant 400 μL of 1.8 N NaOH was added and the optical density was read at 420 nm against the blank (azocasein + TCA + NaOH). The amount of enzyme required to digest 1 mg of azocasein per minute is known as 1 unit of protein activity (Pietrow et al., 2013). The percentage (%) proteolysis was calculated using the following formula:

%Proteolysis=absorbance(sample)−absorbance(blank)/absorbance(⁢control)

### Appraisal of the Therapeutic Challenge of ADM 3 on *S. aureus* Using an *In Vitro* Catheter Model

An *in vitro* catheter model was used to evaluate the effect of ADM 3 on biofilm formation in hydrodynamic conditions (Hancock et al., 2010). Silicone catheter segments of 20 mm length were cut vertically into two halves, sterilized in 0.5% sodium hypochlorite solution followed by washing with sterilized water. The sterilized catheters were placed in a six-well microtitre plate and the surface was coated with human blood plasma by incubation at 37°C for 24 h. Subsequently, the plasma was removed from the wells and the coated catheters were subjected for the establishment of *S. aureus* infection followed by treatment with ADM 3 for 7 days with concentrations of ¼× MBIC_50_, ½× MBIC_50_, MBIC_50_, and 2× MBIC_50_. The plates were incubated at 37°C for 24 h with shaking at 120 rpm. After 24 h of incubation, the medium was changed along with a fresh dosage of the drug each time and this was repeated for 7 days. Also, at every 24 h interval, the catheters from the wells were removed and analyzed for the viability of cells (Weiss et al., 2009). Briefly, the viability of cells from the catheters was processed by immersing the catheters individually into sterile PBS and followed by sonication. The adherent cells recovered in PBS were plated for colony count in TSB agar plates. Microscopy imaging was used to visually analyze the effect of ADM 3 on biofilm removal. The cells were stained with fluorescein isothiocyanate (5 mg/mL) and ethidium bromide (1.25 mg/mL) prepared by mixing 5 μL each of the dyes in 1 mL of cold 0.9% NaCl solution. The samples were incubated for 10 min, and then the excess dye was removed by washing with 0.9% NaCl. All the stained samples were imaged using Olympus FV 1000 confocal microscope with a 10× objective of numerical aperture 0.3. The stained samples were excited at 488 nm using Multi Argon LASER and images were collected from four randomly chosen spots from the sample surface.

### Cytotoxicity Analysis of ADM 3 on HEp-2 Cells

Cell viability was assessed by MTT assay. The reduction of MTT is catalyzed by mitochondrial dehydrogenase enzymes and thus considered to be a measure of cell viability. HEp-2 cells were seeded to each well in a 96-well microtiter plate (1 × 10^5^ cells), in 100 μL of DMEM growth medium and the plate was incubated at 37°C for 24 h. After incubation, the fresh growth medium was replaced in the wells. ADM 3 was diluted from an original stock to obtain a concentration of 200 μg/mL. The HEp-2 cells were incubated with ADM 3 and allowed to adhere for a period of 72 h. A total of 10 μL of MTT solution (5 mg/mL in 1× PBS) was added to each well and the plate was incubated in dark for 4 h at 37°C. Further, the content of the each well was removed and the formazan crystals were dissolved in 200 μL of dimethyl sulfoxide (DMSO) solution per well. The absorbance was measured at 590 nm using ELISA plate reader. Only the interior rows of the microtiter plate were used for these experiments to minimize the variations in cell viability due to medium evaporation at the periphery site. The percentage cell viability was calculated with reference to the untreated control cells. The experiment was conducted twice in quadruplicates.

$$Percentage\,\;cell\,\;viabilty=\frac{\text{OD of drug treated sample-OD of blank}}{\text{OD of control-OD of blank}}\times 100$$

### Statistical Analysis

Graph pad prism software (version 6.01) was used for statistical analysis. One-way ANOVA and multiple comparisons were done. The minimum level of significance was set at *P* ≤ 0.05. All the assays were conducted in triplicates and the results were expressed as mean ± SD.

## Results

### Synthesis of Acyclic Amines and Diamines

The spectral data of all the synthesized compounds were in full agreement with the proposed structures (Supplementary Table S1). The ^1^H-NMR data for the synthesized compounds are given below.

**AAM 1: *N*-(naphthalene-2-ylmethyl)butan-1-amine**

Isolated yield = 78%,^1^H NMR (300 MHz, CDCl_3_) δ 8.12 (d, *J* = 8.3 Hz, 1H), 7.90–7.83 (m, 1H), 7.77 (d, *J* = 7.9 Hz, 1H), 7.59–7.38 (m, 4H), 4.24 (s, 2H), 2.81–2.71 (m, 2H), 1.61 (s, 1H), 1.52 (dd, *J* = 8.2, 6.1 Hz, 2H), 1.38 (dq, *J* = 14.1, 7.1 Hz, 2H), 0.93 (t, *J* = 7.3 Hz, 3H).

**AAM 2: (*N*-benzylbutan-1-amine)**

Isolated yield = 55%, ^1^H NMR (300 MHz, CDCl_3_) δ 7.38–7.19 (m, 5H), 3.79 (s, 2H), 2.63 (t, *J* = 7.2 Hz, 2H), 1.76 (s, 1H), 1.50 (dt, *J* = 14.4, 7.0 Hz, 2H), 1.40–1.24 (m, 2H), 0.91 (t, *J* = 7.2 Hz, 3H).

**AAM 3: (*N*-benzylcyclohexanamine)**

Isolated yield = 71%, ^1^H NMR (300 MHz, CDCl_3_) δ 7.36–7.22 (m, 5H), 3.81 (s, 2H), 2.53–2.44 (m, 1H), 1.31–1.06 (m, 11H).

**AAM 4: 4(*N*-(4-methoxybenzyl)butan-1-amine)**

Isolated yield = 65%, ^1^H NMR (300 MHz, CDCl_3_) δ 7.28 – 7.22 (m, 2H), 6.91 – 6.81 (m, 2H), 3.79 (s, 3H), 3.74 (s, 2H), 2.66 – 2.58 (m, 2H), 2.49 (s, 1H), 1.52 (ddd, *J* = 14.4, 8.3, 5.9 Hz, 2H), 1.34 (dd, *J* = 15.1, 7.4 Hz, 2H), 0.90 (t, *J* = 7.3 Hz, 3H).

**AAM 5: (*N*-(4-chlorobenzyl)butan-1-amine)**

Isolated yield = 73%, ^1^H NMR (300 MHz, CDCl_3_) δ 7.33–7.21 (m, 4H), 3.75 (s, 2H), 2.64–2.57 (m, 2H), 1.68 (s, 1H), 1.55–1.43 (m, 2H), 1.40–1.27 (m, 2H), 0.91 (t, *J* = 7.3 Hz, 3H).

**ADM 1: 2,2^1^-((Ethane-1,2-diylbis(azanediyl)bis(methylene))diphenol**

Yield 72%, ^1^H-NMR (DMSO-d_6_, 300 MHz): δ 2.51 (s, 4H), 3.79 (s, 4H), 6.68–6.73 (m, 4H), 7.03–7.08 (m, 4H).

**ADM 2: 2,2′-((Propane-1,3-diylbis(azanediyl)bis(methylene))diphenol**

Yield 79%, ^1^H-NMR (DMSO-d_6_, 300 MHz): δ 1.63 (quin, *J* = 6.9 Hz, 2H), 2.55 (t, *J* = 6.9 Hz, 4H), 3.80 (s, 4H), 6.67–6.72 (m, 4H), 7.03–7.08 (m, 4H).

**ADM 3: 2,2^1^-((Butane-1,4-diylbis(azanediyl)bis(methylene))diphenol**

Yield 69%, ^1^H-NMR (DMSO-d_6_, 300 MHz): δ 1.47 (s, 4H), 2.50 (q, *J* = 1.8 Hz, 4H), 3.81 (s, 4H), 6.66–6.72 (m, 4H), 7.03–7.09 (m, 4H).

**ADM 4: N^1^,N^2^-dibenzylethane-1,2-diamine**

Yield 82%, ^1^H-NMR (DMSO-d_6_, 300 MHz): δ 2.51 (s, 4H), 3.66 (s, 4H), 7.20–7.32 (m, 10H).

**ADM 5: N^1^,N^2^-dibenzylpropane-1, 3-diamine**

Yield 73%, ^1^H-NMR (CDCl_3_, 300 MHz): δ 1.72 (quin, *J* = 6.9 Hz, 2H), 2.69 (t, *J* = 6.9 Hz, 4H), 3.77 (s, 4H), 7.24–7.32 (m, 10H).

**ADM 6: N^1^,N^2^-bis(naphthalene-2-ylmethyl)ethane-1,2-diamine**

Yield 76%, ^1^H-NMR (DMSO-d_6_, 300 MHz): δ 2.75 (s, 4H), 4.11 (s, 4H), 7.40–7.52 (m, 8H), 7.80 (d, *J* = 7.5 Hz, 2H), 7.89–7.92 (m, 2H), 8.13–8.17 (m, 2H).

**ADM 7: N^1^,N^3^-bis(naphthalene-2-ylmethyl)propane-1,3-diamine**

Yield 78%, ^1^H-NMR (CDCl_3_, 300 MHz): δ 1.61 (t, *J* = 6.3 Hz, 4H), 2.76 (t, *J* = 6.3 Hz, 4H), 4.22 (s, 4H), 7.41–7.55 (m, 8H), 7.76 (d, *J* = 7.5 Hz, 2H), 7.86 (d, *J* = 7.5 Hz, 2H), 8.10 (d, *J* = 7.8 Hz, 2H).

**ADM 8: N^1^-(naphthalene-2-yl)–N^4^-(naphthalene-2-ylmethyl)butane-1,4-diamine**

Yield 80%, ^1^H-NMR (DMSO-d_6_, 300 MHz): δ 1.83 (quin, *J* = 6.9 Hz, 2H), 2.83 (t, *J* = 6.9 Hz, 4H), 4.21 (s, 4H), 7.37–7.52 (m, 8H), 7.76 (dd, *J* = 7.5, 1.8 Hz, 2H), 7.85–7.88 (m, 2H), 8.07–8.11 (m, 2H).

### Antimicrobial and Antibiofilm Activity of Acyclic Amines and Diamines

The MICs, MBCs, and MBICs of acyclic amines and diamines were shown in **Table 2**. In general, acyclic amines were found to have high MIC and MBC values but no antibiofilm activity. Alternatively, the acyclic diamine compounds, particularly ADM 3 was observed to exhibit good antimicrobial as well as antibiofilm activity. The concentration of antimicrobial and antibiofilm activity of ADM 3 was found to be 50 and 5 μg/mL (MBIC_50_) respectively. The biofilm inhibitory concentration for compound ADM 3 was found to be 10-fold lesser than the MIC. Thus, compound ADM 3 acts as a potential antibacterial as well as antibiofilm agent and was considered for further studies.

**Table 2:** Antimicrobial and antibiofilm values of acyclic amines and diamines.

Compound	MIC (μg/mL)	MBC (μg/mL)	MBIC (μg/mL)	
			
			50	90
AAM 1	200	200	–	–
AAM 2	200	200	–	–
AAM 3	–	–	–	–
AAM 4	100	200	–	–
AAM 5	–	200	–	–
ADM 1	200	200	–	–
ADM 2	–	50	10	–
ADM 3	50	200	5	–
ADM 4	–	–	25	–
ADM 5	–	200	20	–
ADM 6	–	–	40	–
ADM 7	–	–	40	–
ADM 8	150	–	–	–


### Anti-virulence Assays

**FIGURE 2:**
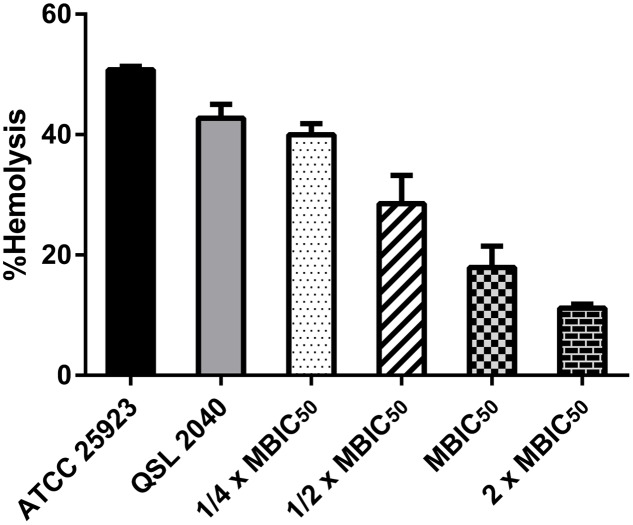
Effect of ADM 3 on hemolysin production in *S. aureus* (ATCC 25923—reference strain; QSL2040—clinical isolate). The concentrations used were ¼× MBIC_50_ (1.25 μg/mL), ½× MBIC_50_ (2.5 μg/mL), MBIC_50_ (5 μg/mL), and 2× MBIC_50_ (10 μg/mL).

**FIGURE 3:**
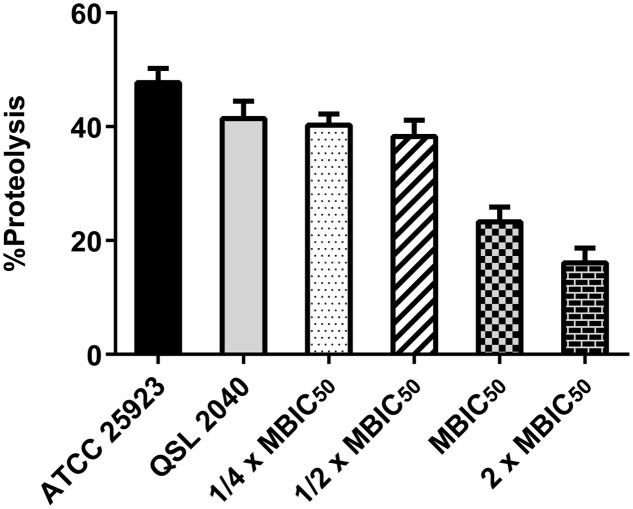
Effect of ADM 3 on protease production in *S. aureus* (ATCC 25923—reference strain; QSL2040—clinical isolate). The concentrations used were ¼× MBIC_50_ (1.25 μg/mL), ½× MBIC_50_ (2.5 μg/mL), MBIC_50_ (5 μg/mL), and 2× MBIC_50_ (10 μg/mL).

Percentage hemolysis and proteolysis by ADM 3 on *S. aureus* clinical isolate, MDRSA - QSL2040 are shown in **Figures 2**, **3** respectively. ADM 3 exhibited a similar effect on protease as well as on hemolysin production and a dose-dependent reduction was observed in both the cases. Clinical isolate (MDRSA - QSL2040) treated with ADM 3 showed a significant reduction in proteolytic and hemolytic activity when compared with the untreated control.

### *In Vitro* Catheter Model

**FIGURE 4:**
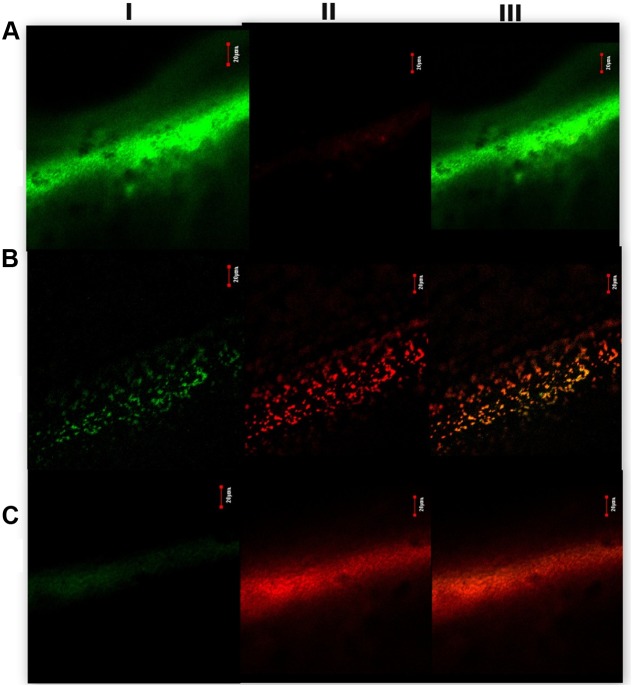
Confocal laser scanning microscope images of biofilm formed on urinary catheters. I—FITC labeled (green/viable cells); II—ethidium bromide labeled (red/non-viable cells); and III—superimposed images of I and II. **(A)** Control, untreated with ADM 3, **(B)** treated with ADM 3 (MBIC_50_ at 5 μg/mL), and **(C)** treated with ADM 3 (2× MBIC_50_ at 10 μg/mL).

Live dead staining of *S. aureus* in the presence and absence of ADM 3 revealed that, ADM 3 treatment induced significant loss of viability and colonization of biofilm on catheters as evidenced by the red fluorescence observed in treated samples at day 7. All the captured images were uniform and a representative image is shown in **Figure 4**. Thus, in consonance with our antibiofilm results in microtitre plate experiments, ADM 3 was effective in inhibition of *S. aureus* biofilm in catheters. The treatment of these catheters showed a decrease in the cell count from day 4 onward compared to untreated control (Supplementary Figure S1).

### Cytotoxicity Analysis on HEp-2 Cell Lines

**FIGURE 5:**
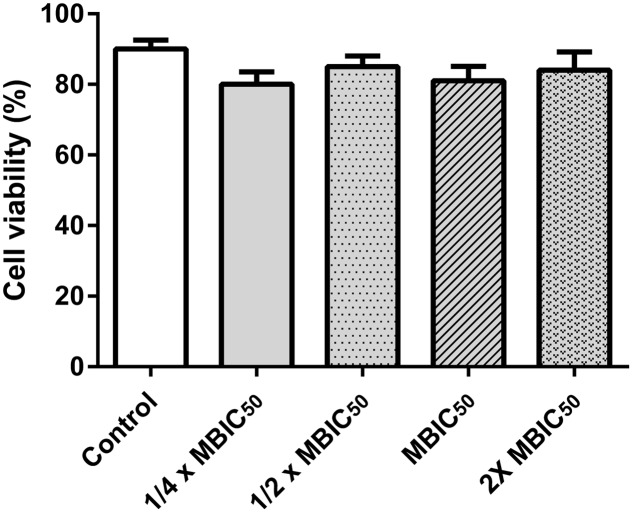
Effect of ADM 3 on HEp-2 cell lines for cell viability by MTT assay. The concentrations used were ¼× MBIC_50_ (1.25 μg/mL), ½× MBIC_50_ (2.5 μg/mL), MBIC_50_ (5 μg/mL), and 2× MBIC_50_ (10 μg/mL).

Percentage cell viability of each experimental group was calculated with reference to the untreated control cells and it was observed that cell viability was higher than 85% in all drug doses (**Figure 5**). Statistical analysis was performed using the one way ANOVA and *P* ≤ 0.001 was considered significant.

## Discussion

The key consequences of biofilm based infections are resistant to antibiotics as well as many other conventional antimicrobial agents. This eventually leads to the development of MDR pathogens with an extreme capacity for evading the host defense mechanisms. It is well known that bacterial biofilm exhibits antibiotic diffusion barrier (Cloete, 2003). In such cases, a large portion of the cells embedded in biofilms might escape from both antibiotic treatments as well as host’s immune system responses. Such bacterial cells lead to the replenishment of biofilm at a new site causing are occurrence of infection with high resistance to antibiotics. Consequently, it becomes difficult to treat the infection using bactericidal drugs via systemic administration and demands the need for the evaluation of novel antibiofilm drugs. As an alternative to conventional antibiotic therapy and to control MDR, we hereby report a series of acyclic amines and diamines as potential drug candidates in the light of our earlier studies (Arya and Princy, 2013; Balamurugan et al., 2017). Among these, ADM 3 (2,2′-((butane-1,4-diylbis(azanediyl)bis(methylene))diphenol) showed a significant reduction in bacterial cell count as well as biofilm inhibition.

Targeting virulence factors in bacteria is an alternative approach to antimicrobial therapy that offers promising insights and opportunities to inhibit bacterial pathogenesis (Kauppi et al., 2003; Åberg and Almqvist, 2007). Certain virulence factors have been shown as potential leads for drug design and therapeutic intervention, whereas new possible insights are crucial for exploring others (Projan, 2002). Among the various virulence factors expressed in *S. aureus*, protease, as well as hemolysin production, plays a crucial role in the interaction of bacteria with the host cell for establishing an infection (Kupferwasser et al., 2003). In the present study, ADM 3 that showed effective MBIC_50_ and MIC was also effective in reducing protease as well as hemolysin production.

*In vitro* catheter model showed decreased biofilm as well as increased red fluorescence in the confocal laser scanning microscope images at day 7. This suggests that ADM 3 has both antimicrobial and antibiofilm activities. In addition, decreased cell count observed after day 4 till the experimental study period also suggests the potential of ADM 3. This implies that the ADM 3 was proven to show a potent biofilm inhibiting agent as well as an antimicrobial agent. It is already shown through our *in silico* studies that the hydroxyl and amine groups of the compound SarABI-12, interact with the oxygen atoms of E89 and R90 of SarA protein respectively, to form hydrogen bonds (Arya and Princy, 2013). Moreover, our *in vitro* studies also have confirmed the antibiofilm activities by the target specific interaction with SarA, which is a quorum regulator of *S. aureus*. Since ADM 3 is a derivative of SarABI-12, which carries both the hydroxyl and amine groups, it is speculated that the biofilm inhibition activity also could be of the similar mechanism.

Furthermore, a therapeutic molecule should not have cytotoxic effects if it has to be taken for further clinical trials. Generally, a compound is usually considered to have *in vitro* cytotoxicity if the particular concentration of the drug causes a 50% cell killing. Our data suggest that the compound ADM 3 may act as a potential drug which does not cause any cytotoxicity. To the best of our knowledge, our study is the first to report the antimicrobial and antibiofilm activity of acyclic amines and diamines. Our data also provide an insight that these compounds can act as a potential drug candidate to treat MDRSA. It would be interesting to explore the activity of these organic molecules at the molecular level to depict their mode of action involved in antibiofilm and antibacterial activity.

## Author Contributions

All authors listed have made a substantial, direct and intellectual contribution to the work, and approved it for publication.

## Conflict of Interest Statement

The authors declare that the research was conducted in the absence of any commercial or financial relationships that could be construed as a potential conflict of interest.
