# Supplementary material for: Antimicrobial and Antibiofilm Potential of Acyclic Amines and Diamines against Multi-Drug Resistant Staphylococcus aureus
Source: Front Microbiol. 2017 Sep 15;8:1767. doi: 10.3389/fmicb.2017.01767 (PMC5605668; doi:10.3389/fmicb.2017.01767)
Supplement: Supplementary file 1 [file Data_Sheet_1.DOCX]

**Supplementary Information**

Antimicrobial and antibiofilm potential of acyclic amines and diamines against multidrug resistant *Staphylococcus aureus*

**Table S1** Reactants for the synthesis of acyclic amines and diamines and their corresponding structures

| **Annotation** | **Aryl Aldehyde** | **Primary / Secondary Amine** | **Compound Name / Structure** |
| --- | --- | --- | --- |
| AAM 1 | Naphthaldehyde | Butylamine |  |
| AAM 2 | Benzaldehyde | Butylamine |  |
| AAM 3 | Benzaldehyde | Cyclohexylamine |  |
| AAM 4 | 4-methoxy benzaldehyde | Butylamine |  |
| AAM 5 | 4-chloro benzaldehyde | Butylamine |  |
| ADM1 | Salicylaldehyde | Ethylenediamine |  |
| ADM 2 | Salicylaldehyde | 1,3-Diaminopropane |  |
| ADM 3 | Salicylaldehyde | 1,4-Diaminobutane |  |
| ADM 4 | Benzaldehyde | Ethylenediamine |  |
| ADM 5 | Benzaldehyde | 1,3-Diaminopropane |  |
| ADM 6 | Naphthaldehyde | Ethylenediamine |  |
| ADM 7 | Naphthaldehyde | 1,3-Diaminopropane |  |
| ADM 8 | Naphthaldehyde | 1,4-Diaminobutane |  |

**
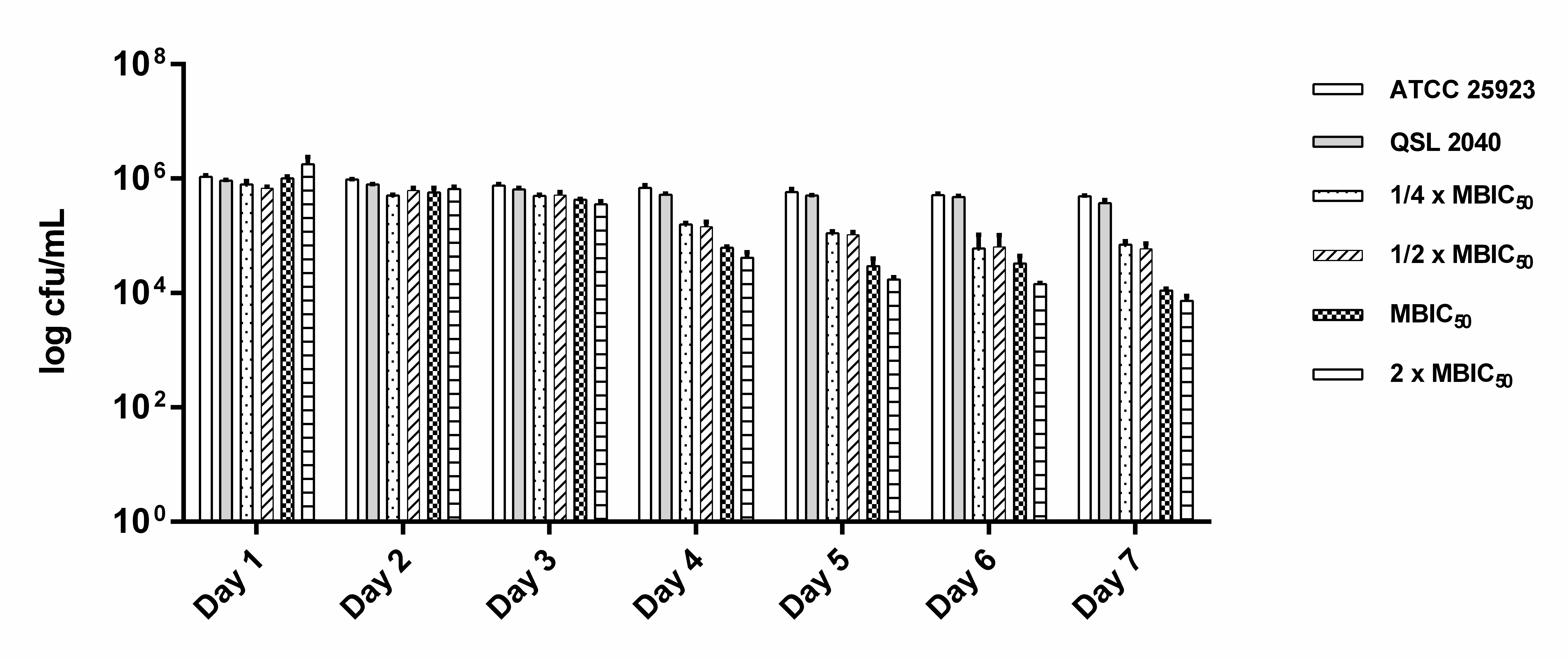
**

**Figure S1** Effect of ADM 3 on the viability of *S. aureus* over seven days of treatment with and without drug. The concentrations used were (¼ X MBIC_50_ (1.25 μg/mL), ½ X MBIC_50_ (2.5 μg/mL), MBIC_50_ (5 μg/ mL) and 2X MBIC_50_ (10 μg/mL). (ATCC 25923 (reference strain); QSL 2040- clinical isolate)
